# From Signal to Symptom: EEG Paroxysms and Background Slowing as Potential Biomarkers and Compensatory Failures in Treatment-Resistant Schizophrenia

**DOI:** 10.3390/biomedicines14030641

**Published:** 2026-03-12

**Authors:** Georgi Panov, Presyana Panova, Silvana Dyulgerova, Ivan Chakarov

**Affiliations:** 1 Psychiatric Clinic, University Hospital for Active Treatment “Prof. Dr. Stoyan Kirkovich”, Trakia University, 6000 Stara Zagora, Bulgaria; 2 Medical Faculty, University “Prof. Dr. Asen Zlatarov”, 8000 Burgas, Bulgaria; 3 Medical Faculty, Trakia University, 6000 Stara Zagora, Bulgaria; 4 Department of Pediatrics, Faculty of Medicine, Trakia University, 6000 Stara Zagora, Bulgaria; 5 Department of Urology, Children Urology, UMHATEM ‘N.I.Pirogov’, 1606 Sofia, Bulgaria

**Keywords:** treatment-resistant schizophrenia, electroencephalography, interictal epileptiform discharges, biomarkers, cortical excitability, kynurenine pathway, forced normalization, neurophysiology, psychosis, default mode network

## Abstract

Background: Schizophrenia is a heterogeneous disorder, and treatment-resistant schizophrenia (TRS) affects 20–30% of patients, yet objective biomarkers for its identification remain limited. Routine electroencephalography (EEG) offers a non-invasive window into cortical network dynamics, with previous studies reporting paroxysmal epileptiform activity and background slowing in a subset of patients. However, the biological significance of these findings—whether purely pathological or potentially compensatory—remains unclear. This study aimed to compare EEG abnormalities between TRS patients and those in clinical remission and to propose an integrative neurobiological interpretation. Methods: In a cross-sectional design, 89 patients with schizophrenia (39 TRS, 50 in remission) underwent routine EEG recordings using the international 10–20 system. TRS was defined according to TRRIP consensus criteria, requiring <20% symptom reduction after adequate antipsychotic trials. EEG analysis focused on the prevalence of interictal epileptiform discharges (IEDs) and the severity of background slowing, assessed on a 4-point ordinal scale. Results: IEDs were more than twice as prevalent in TRS patients compared to those in remission. Background slowing was significantly more severe in the TRS group, with the majority showing moderate-to-severe abnormalities versus predominantly normal-to-mild patterns in remission patients. Focal EEG abnormalities also followed this pattern. Multivariate analysis confirmed that both IEDs and background severity were independent predictors of TRS. Conclusions: EEG abnormalities, particularly IEDs and background slowing, are potential neurophysiological signatures associated with treatment resistance. We propose an integrative hypothesis suggesting that IEDs may originate as a failed compensatory mechanism—the brain’s attempt to restore network homeostasis. In chronic TRS these discharges become maladaptive, contributing to excitotoxicity and network dysfunction. This framework opens avenues for EEG-based stratification and novel therapeutic strategies targeting cortical excitability.

## 1. Introduction

Schizophrenia is a severe and heterogeneous psychiatric disorder whose etiology arises from a complex interplay of genetic susceptibility and epigenetic modifications influenced by environmental factors [1,2,3,4,5]. This multifactorial pathogenesis translates into a diverse clinical presentation, encompassing the core dimensions of positive, negative, and cognitive symptoms [6,7,8,9], frequently accompanied by comorbid affective, anxiety, and obsessive-compulsive features [10,11,12]. The global burden of schizophrenia remains substantial, with significant implications for individual functioning, healthcare systems, and society at large [13].

A significant portion of patients—approximately 20–30%—develop treatment-resistant schizophrenia (TRS), defined by a persistent lack of response to adequate trials of at least two antipsychotic drugs [14]. This clinical challenge underscores the critical need for objective biomarkers that can aid in early identification, stratification, and pathophysiological understanding of TRS. While the diagnosis of schizophrenia remains primarily clinical, considerable research efforts have been directed toward identifying biological correlates. Neuroimaging techniques, though informative, often lack the specificity and accessibility required for routine clinical stratification. In contrast, routine electroencephalography (EEG) offers a non-invasive, widely available window into cortical network dynamics.

Characteristic EEG alterations in schizophrenia include generalized slowing of background rhythms—manifesting as increased theta and delta power alongside reduced alpha activity—which reflects widespread cortical dysfunction and impaired thalamocortical regulation [15,16,17]. Beyond these diffuse abnormalities, a subset of patients exhibits paroxysmal EEG activity, comprising sharp waves, spikes, and epileptiform discharges—referred to as interictal epileptiform discharges (IEDs). This phenomenon has been consistently linked to greater symptom severity, cognitive deficits, and a poorer response to antipsychotic medication, positioning it as a potential biomarker for TRS [10,18,19].

However, the neurobiological interpretation of IEDs remains a subject of debate. A purely pathophysiological view frames it as a marker of disrupted excitatory-inhibitory (E/I) balance and network instability. Conversely, an emerging perspective suggests that such transient, synchronized discharges might also represent an adaptive, albeit maladaptive in its chronic form, attempt by the brain to restore functional homeostasis in the face of pervasive dysregulation [19,20,21]. This conceptual duality invites an exploration of underlying mechanisms, yet the literature lacks an integrative model that explains the biological purpose—whether pathological or compensatory—of these electrophysiological phenomena.

The tryptophan-kynurenine metabolic pathway (KP) has emerged as a critical regulator of brain excitability and neuroinflammation. In schizophrenia, a dysregulated KP is characterized by an imbalance between neuroprotective (kynurenic acid, KYNA) and neurotoxic (quinolinic acid, QUIN) metabolites, directly influencing NMDA receptor function and contributing to both E/I imbalance and oxidative stress [22,23,24,25,26,27,28]. Intriguingly, controlled neuronal hyperexcitation, as induced by electroconvulsive therapy (ECT), has been shown to modulate the KP toward a more neuroprotective state, suggesting a link between synchronized neuronal discharges and metabolic adaptation [29,30,31]. This raises a provocative question: could spontaneous, pathological ***IEDs*** in schizophrenia engage similar neurochemical pathways, initially serving a failed compensatory purpose before evolving into a source of further pathology?

To address this gap, we conducted a cross-sectional EEG study comparing patients with TRS to those in clinical remission. Our primary objectives were: (1) to quantify and compare the prevalence of IEDs and the severity of background abnormalities between the two groups, and (2) to interpret these findings within an integrative neurobiological framework that considers both the KP dysregulation and the potential dual—pathological versus compensatory—nature of paroxysmal electrophysiological events in schizophrenia. By bridging clinical neuroscience with metabolic hypotheses, this study aims to contribute to biomarker-driven stratification and future therapeutic innovation.

## 2. Methods

This cross-sectional study was designed to investigate neurophysiological differences between patients with treatment-resistant schizophrenia and those in clinical remission using standardized routine EEG recordings.

### 2.1. Participants

A total of 89 patients with a diagnosis of schizophrenia were included in this cross-sectional study. Participants were recruited from the inpatient and outpatient services of the Psychiatric Clinic at Stara Zagora University Hospital following admission for acute psychotic episodes. The cohort comprised 58 females and 31 males. Patients were prospectively enrolled and followed between 2017 and 2022. The present analysis utilizes the same core cohort from a larger, ongoing research project on clinical and neurophysiological markers in schizophrenia, parts of which have been previously published, focusing on other clinical dimensions such as cognition, obsessive-compulsive symptoms, dissociation, lateralization, gender differences, depressive features, and the role of untreated psychosis [32,33,34,35,36,37,38,39,40].

Diagnosis was confirmed according to ICD-10 and DSM-5 criteria [41,42,43]. Patients were subsequently classified into one of two groups based on their treatment response: Treatment-Resistant Schizophrenia (TRS) or Schizophrenia in Clinical Remission.

The classification of Treatment Resistance followed the principles outlined by the Treatment Response and Resistance in Psychosis (TRRIP) working group consensus [14,44]. Specifically, patients were included in the TRS group if they met all of the following criteria:

1. Prospective observation for at least 12 weeks.
2. Administration of at least two different antipsychotic drug trials at a dose equivalent to or greater than 600 mg of chlorpromazine.
3. A reduction of less than 20% in symptom severity scores on both the Positive and Negative Syndrome Scale (PANSS) [45] and the Brief Psychiatric Rating Scale (BPRS) [46] during the observation period.
4. A score below 60 on the Social and Occupational Functioning Assessment Scale (SOFAS), indicating significant social dysfunction.

Patients were classified as being in Clinical Remission if they met the standardized, consensus-based criteria for remission in schizophrenia, requiring a sustained period of low symptom intensity on core PANSS items [47].

Exclusion criteria for all participants were: (1) intellectual disability; (2) active substance abuse disorder; (3) documented organic brain damage; (4) concomitant progressive neurological or severe somatic illness; (5) pronounced personality change (assessed via DSM-5/ICD-10 criteria) [41,42]; (6) a score below 25 on the Mini-Mental State Examination (MMSE); and (7) pregnancy or lactation. An additional practical criterion was the patient’s ability to cooperate during the EEG recording, requiring a calm, relaxed state and the capacity to follow simple commands.

### 2.2. EEG Recording and Analysis

All participants underwent a routine EEG recording performed in accordance with international standards [48,49]. The examination was conducted in a quiet, dimly lit room with the patient seated in a comfortable, reclining chair. A digital EEG system (WIN-EEG, version 2.138.111(02.2020), Mitsar-EEG, Mitsar Ltd, St Petersburg) with electrodes placed according to the international 10–20 system was used, ensuring electrode impedances remained below 5 kΩ. EEG recordings were performed using the international 10–20 system with 21 silver chloride (Ag/AgCl) electrodes. The reference electrode was placed at the vertex (Cz), and the ground electrode was placed on the forehead (Fpz). Both longitudinal (bipolar) and transverse montages were used for the detection and localization of epileptiform activity. The standard recording protocol included consecutive periods of rest with eyes closed and eyes open, hyperventilation (3–5 min) in the absence of contraindications, and intermittent photic stimulation. The minimum duration of interpretable brain activity recording was 20 min, in accordance with international standards [48,49].

The analysis focused on two primary categories of EEG activity:

1. Interictal Epileptiform Discharges (IEDs):

Identification of ***IEDs*** was based on established morphological criteria [48,50,51]: (a) clear distinction from the background activity in terms of morphology, frequency, and amplitude; (b) the presence of a sharp configuration (spike: 20–70 ms, or sharp wave: 70–200 ms), often followed by a slow wave of opposite polarity; and (c) demonstration of a physiological electrical field with a logical distribution and amplitude gradient across adjacent electrodes. Supporting features such as abrupt onset/offset, tendency to occur in rhythmic sequences or bursts, and activation during provocation (hyperventilation, photic stimulation, sleep) were also noted. Activity was categorized as focal or generalized.

2. Background Activity:

The background rhythm was assessed by comparison with age-adjusted norms [51]. Abnormalities were categorized on a 4-point ordinal severity scale based on the degree of slowing, disorganization, and loss of reactivity: (1) Normal—age-appropriate posterior dominant rhythm with good organization; (2) Mild impairment—mild excess of diffuse theta activity with preserved alpha frequency and reactivity; (3) Moderate impairment—mixed theta and delta activity significantly obscuring or replacing the background rhythm, with reduced organization and reactivity; (4) Severe impairment—dominant, high-amplitude diffuse delta/theta activity with no organized posterior alpha rhythm and absence of normal reactivity.

All EEG records were reviewed independently by an experienced clinical neurophysiologist.

### 2.3. Statistical Analysis

Statistical analysis was performed using SPSS software (version 26). Descriptive statistics were calculated for demographic and clinical variables. Given the ordinal nature of the EEG background scale and non-normal distribution of some data, non-parametric tests were employed. Group differences (TRS vs. Remission) in the prevalence of IEDs and focal abnormalities were assessed using the Chi-square test or Fisher’s exact test, as appropriate. The Mann–Whitney U test was used to compare the ordinal EEG background scores between groups. To determine the independent contribution of EEG variables to treatment resistance, a binary logistic regression analysis was performed, with group (TRS vs. Remission) as the dependent variable. The model included *IEDs* (present/absent), background severity score, age of onset, illness duration, and gender as predictors. Correlation analyses were conducted to explore relationships between variables. A *p*-value of less than 0.05 was considered statistically significant.

### 2.4. Ethical Considerations

The study was conducted in accordance with the ethical principles of the Declaration of Helsinki. The study protocol was reviewed and approved by the Ethical Committee of the University Hospital “Prof. Dr. Stoyan Kirkovich” in Stara Zagora (Protocol Code: TR3--02-242, Date: 30 December 2021). All participants, or their legal guardians, provided written informed consent after receiving a detailed explanation of the study procedures, its safety, and the confidentiality of the obtained data.

## 3. Results

### 3.1. Demographic and Clinical Characteristics of the Sample

The study included 89 patients with schizophrenia, classified into a treatment-resistant group (TRS, *n* = 39) and a remission group (*n* = 50). As summarized in Table 1, the two groups were well-matched on key demographic parameters. There were no significant differences in age, height, weight, or Body Mass Index (BMI) between patients with TRS and those in remission (*p* > 0.05 for all). However, as expected and consistent with the natural history of the disorder and our previous findings from this cohort [32,40,52], the age of onset was significantly earlier in the TRS group (23.33 ± 7.31 years) compared to the remission group (27.52 ± 8.45 years; *p* = 0.018). Consequently, the duration of schizophrenia was significantly longer in the TRS group (13.69 ± 11.45 years) than in the remission group (9.14 ± 6.98 years; *p* = 0.016).

### 3.2. Prevalence of Interictal Epileptiform Discharges (IEDs)

**Figure 1:**
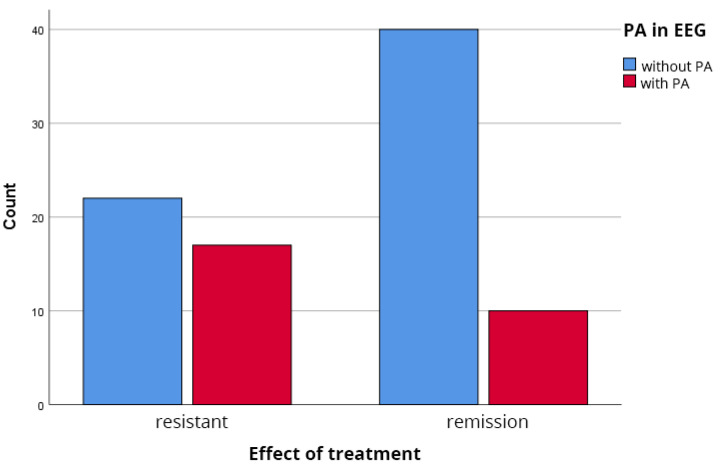
Prevalence of interictal epileptiform discharges (IEDs) in the two groups. TRS group (*n* = 39) showed 43.6% prevalence vs. Remission group (*n* = 50) with 20.0% prevalence. Chi-square test: χ^2^(1) = 5.77, *p* = 0.016; Fisher’s exact test *p* = 0.021.

The overall prevalence of IEDs in the entire sample was 30.3% (27 of 89 patients). However, its distribution was highly unequal between the clinical groups (Figure 1). IEDs were identified in 17 of the 39 patients with TRS (43.6%), compared to only 10 of the 50 patients in remission (20.0%). This difference was statistically significant (χ^2^(1) = 5.77, *p* = 0.016; Fisher’s exact test *p* = 0.021). The odds ratio of 0.32 (95% CI: 0.12–0.83) indicates that patients in remission had approximately 68% lower odds of exhibiting IEDs compared to patients with TRS.

In patients with IEDs, the activity was predominantly observed in temporal and frontal regions, most commonly involving electrodes F7, F8, T3, T4, F3, and F4. Individual lateralization was observed (13 patients with left-sided, 10 with right-sided, and 4 with bilateral independent activity). Interestingly, in the overall sample of patients with IEDs (*n* = 27), there was a trend toward left hemispheric predominance, with 48.1% showing left-sided activity. Although not statistically significant in our sample due to the small number of patients in each subgroup, this leftward trend is consistent with previous studies suggesting that the left hemisphere may be more prone to epileptogenesis [53,54,55]. The distribution of lateralization was similar between the TRS and remission groups. 

### 3.3. Severity of Background EEG Abnormalities

**Figure 2:**
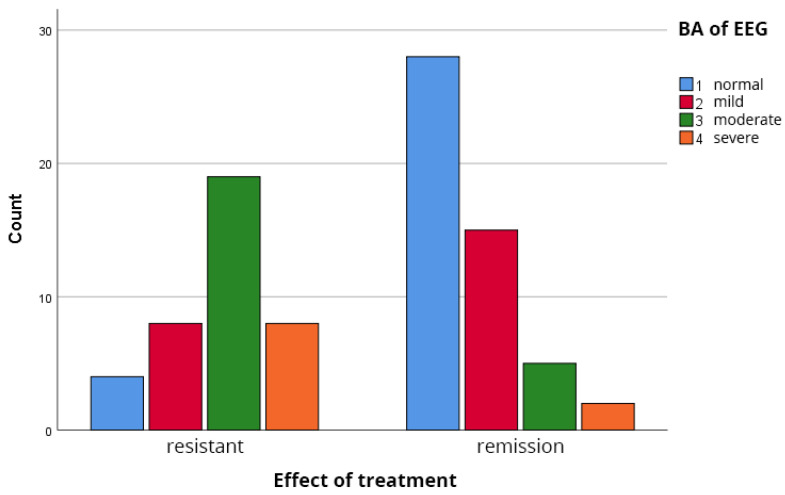
Distribution of the changes of the Background EEG Activity in the groups. Mann–Whitney U test: *p* < 0.001, effect size r = 0.55.

The analysis of background EEG rhythms revealed a pronounced difference between the groups. As shown in Table 2, Figure 2, the majority of patients in remission (86.0%) displayed normal or only mildly abnormal backgrounds. In complete contrast, the TRS group was characterized by moderate to severe background abnormalities, with 69.2% of patients falling into these categories. The median severity score was 1.5 (IQR: 1–2) for the remission group and 3.0 (IQR: 3–4) for the TRS group. This difference was highly statistically significant (Mann–Whitney U test, *p* < 0.001), with a large effect size (r = 0.55).

### 3.4. Focal EEG Abnormalities

**Figure 3:**
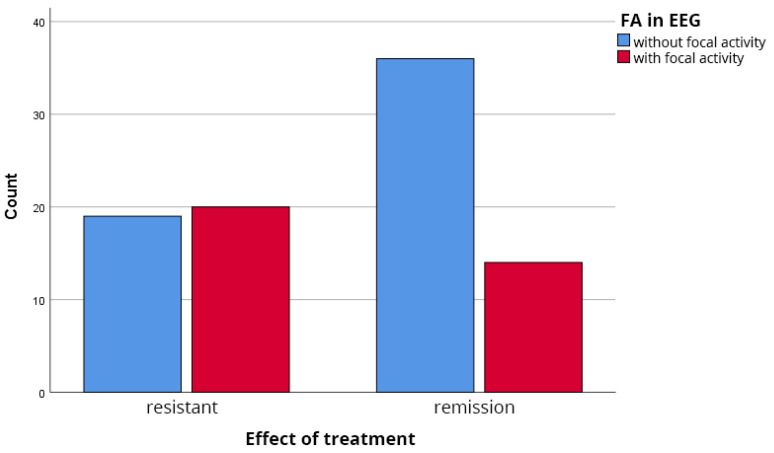
Prevalence of focal EEG abnormalities in the analyzed groups. Chi-square test: χ^2^(1) = 5.03, *p* = 0.025.

Focal EEG abnormalities (slowing or epileptiform activity confined to a specific region) were also significantly more common in the TRS group. They were present in 20 of 39 TRS patients (51.3%) versus 14 of 50 remission patients (28.0%) (χ^2^(1) = 5.03, *p* = 0.025), as shown in Figure 3.

### 3.5. Gender Distribution of EEG Findings

**Figure 4:**
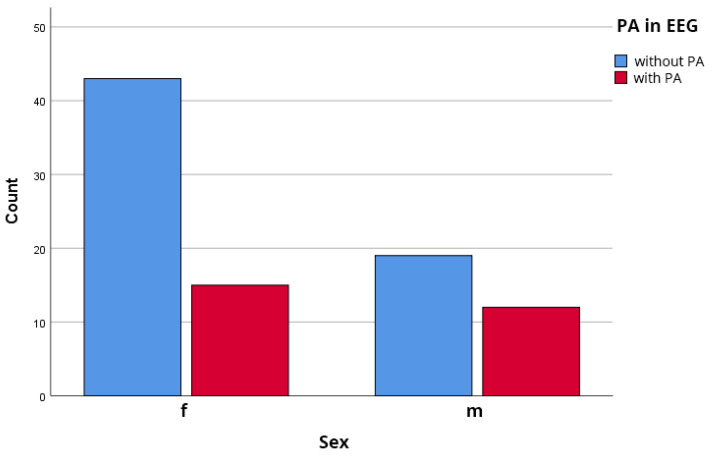
Gender associated distribution of ***IEDs*** on EEG. Males (*n* = 31): 38.7%; Females (*n* = 58): 25.9%. Chi-square test: χ^2^(1) = 1.66, *p* = 0.198 (non-significant).

The sample consisted of 58 females (65.2%) and 31 males (34.8%). The prevalence of ***IEDs*** was numerically higher in males (38.7%, 12/31) than in females (25.9%, 15/58), but this difference did not reach statistical significance (χ^2^(1) = 1.66, *p* = 0.198), as illustrated in Figure 4.

### 3.6. Gender Associated Distribution of the Changes of the Background Activity of the EEG

**Figure 5:**
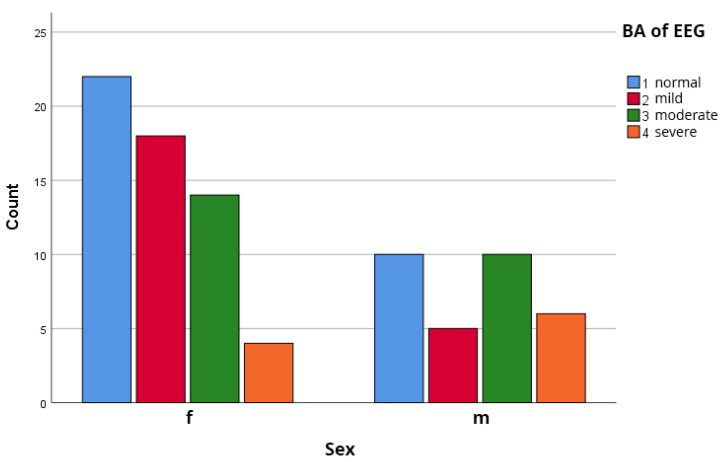
Gender Distribution of Background EEG Activity Findings. Males (*n* = 31): median score 3.0 (IQR: 2–3); Females (*n* = 58): median score 2.0 (IQR: 1–2). Mann–Whitney U test: *p* = 0.069 (trend toward significance).

Analysis of the changes of the background activity revealed a trend toward more pronounced abnormalities in male patients. The median background score was 3.0 (IQR: 2–3) for males and 2.0 (IQR: 1–2) for females (Mann–Whitney U test, *p* = 0.069). Severe background abnormalities (Grade 4) were almost three times more frequent in males (19.4%) than in females (6.9%). These findings align with our previous investigation of gender-associated clinical features in this cohort [32,36,40], as shown in Figure 5.

### 3.7. Multivariate Analysis

To assess whether the EEG findings were independently associated with TRS after accounting for potential confounders, we performed a binary logistic regression analysis. The model, which included *IEDs*, background severity score, age of onset, illness duration, and gender, was statistically significant (χ^2^(5) = 34.2, *p* < 0.001). The analysis revealed that both the presence of *IEDs* (Odds Ratio [OR] = 3.2, 95% Confidence Interval [CI]: 1.1–9.5, *p* = 0.035) and a higher background severity score (OR = 4.1, 95% CI: 2.1–7.8, *p* < 0.001) remained significant independent predictors of belonging to the TRS group. Among the covariates, only illness duration showed a trend toward significance (OR = 1.1, 95% CI: 1.0–1.1, *p* = 0.06), while age of onset (*p* = 0.20) and gender (*p* = 0.15) were not significant predictors in this model (Table 3)

In summary, patients with TRS demonstrated significantly higher prevalence of interictal epileptiform discharges, more severe background slowing, and more frequent focal abnormalities compared to patients in remission. These findings remained significant after controlling for potential confounders in multivariate analysis, indicating that EEG abnormalities are independently associated with treatment resistance.

## 4. Discussion

The present study provides clear neurophysiological evidence differentiating patients with treatment-resistant schizophrenia from those in clinical remission. Our key findings—a significantly higher prevalence of interictal epileptiform discharges and markedly greater severity of background slowing in TRS patients—corroborate and extend previous research linking EEG dysregulation to poor clinical outcomes [18,19,21]. These findings remained significant after controlling for potential confounders, underscoring their robustness as potential biomarkers. However, beyond confirming these associations, our results invite a deeper question: what is the biological meaning of these electrophysiological abnormalities? Are they merely pathological epiphenomena, or might they reflect an underlying adaptive—albeit failed—process? In this discussion, we first interpret our findings within the framework of network instability and excitatory-inhibitory imbalance, then propose a speculative hypothesis regarding the dual-faced nature of paroxysmal activity, and finally explore clinical implications and future directions.

### 4.1. Interictal Epileptiform Discharges as a Marker of Network Instability in TRS

The nearly 2.2-fold higher odds of IEDs in our TRS cohort solidify its role as a potential biomarker of severity and pharmacological resistance. This aligns with studies suggesting that epileptiform discharges in schizophrenia reflect a fundamental breakdown in cortical inhibition and a state of heightened network excitability [19,20]. Such hyperexcitability may be a common neurophysiological endpoint for various pathological processes in TRS, including the dysregulation of the tryptophan-kynurenine pathway (KP). In its neurotoxic state, the KP shifts towards the production of quinolinic acid (QUIN), a potent NMDA receptor agonist, while the levels of the neuroprotective NMDA antagonist kynurenic acid (KYNA) may be relatively deficient [24,25,26]. This QUIN/KYNA imbalance lowers the seizure threshold and promotes the synchronized neuronal bursts observed as ***IEDs*** on EEG [26,56]. Therefore, in established TRS, IEDs likely signify a maladaptive state of chronic excitotoxicity and glutamatergic dysregulation, directly contributing to symptom persistence and treatment failure [57].

These findings align with contemporary models of schizophrenia as a disorder of large-scale brain network dysregulation [58,59,60,61]. The hyperexcitability reflected in *IEDs* may disrupt the normal oscillatory dynamics within key networks such as the default mode network (DMN) and fronto-parietal networks, contributing to the cognitive disorganization and negative symptoms characteristic of TRS. Recent work by Battaglia and colleagues has emphasized how excitatory-inhibitory imbalance can propagate through large-scale cortical networks, leading to widespread dysregulation of neural oscillations and cognitive deficits [60]. Specifically, their research demonstrates that localized disruptions in E/I balance can have cascading effects on network-level dynamics, potentially explaining how focal *IEDs* might contribute to the diffuse background slowing observed in our TRS patients [61].

### 4.2. Background Slowing: A Correlate of Diffuse Dysfunction and Cognitive Impairment

The strong association between treatment resistance and moderate-to-severe background slowing underscores the presence of widespread cortical inefficiency in TRS. This pattern, characterized by an excess of low-frequency (theta/delta) rhythms and disorganization of the posterior alpha rhythm, indicates impaired thalamocortical drive and deficient neural synchrony [15,16,17]. Such diffuse dysfunction likely forms the neurophysiological substrate for the profound cognitive deficits and negative symptoms that are hallmarks of TRS and are strongly associated with poor functional outcomes, as observed in our previous analyses of this cohort [32,35,40]. This abnormal background may create a permissive environment where focal hyperexcitability and IEDs can more easily emerge.

### 4.3. The Dual-Faced Nature of Interictal Epileptiform Discharges: A Hypothesis for a Failed Compensatory Mechanism

While our data firmly establish IEDs as a marker of resistance, the cross-sectional design precludes causal inferences about its origin and evolution. However, building on our findings and existing literature, we propose a speculative hypothesis concerning its potential biological purpose, which requires validation in longitudinal studies. We propose a temporal and contextual duality in the role of IEDs.

In the acute or prodromal phase of network dysregulation, it is plausible that a single, isolated IED might transiently function as a weak, endogenous analog to electroconvulsive therapy (ECT). Both ECT and IEDs are intense, synchronized neuronal events. Notably, ECT has been shown to modulate the KP, increasing KYNA levels and shifting the balance toward neuroprotection [29,30,31]. It is plausible that an initial, pathological discharge could trigger a similar, transient neurochemical response—a brief surge in KYNA or a reset of neuronal membrane potentials—as part of a homeostatic, last-resort attempt to counteract cortical hypoactivity or destabilized network dynamics [20,21]. This speculative “hormetic” response could be viewed as the brain’s intrinsic, albeit crude, effort at self-correction.

However, we hypothesize that in chronic, treatment-resistant schizophrenia, this fragile compensatory mechanism fails and becomes maladaptive. The persistent neuroinflammatory milieu, a key feature of TRS [62], drives the KP into a sustained QUIN-predominant state. In this context, recurrent *IEDs* are no longer a corrective reset but a symptom and a driver of the underlying excitotoxicity. It contributes to neuronal exhaustion, oxidative stress, and maladaptive plasticity (e.g., through kindling-like effects), thereby reinforcing the very network instability it might have initially sought to oppose [20,26]. The severe background slowing observed in our TRS group is a likely electrophysiological signature of this neuronal exhaustion and loss of functional reserve.

This speculative hypothesis finds intriguing parallels in the field of epilepsy, particularly treatment-resistant epilepsy (TRE). The prevalence of IEDs in adults with epilepsy is substantial, with initial routine EEG detecting them in approximately 50% of cases, a figure that increases with repeated or prolonged monitoring [63]. This range is strikingly similar to the 43.6% prevalence of IEDs we observed in TRS. Furthermore, a comparable therapeutic challenge exists at the very onset of treatment: seminal work in epilepsy has shown that about 47% of patients achieve remission with their first appropriately chosen antiepileptic drug [64]. Our analysis within the present schizophrenia cohort aligns with this pattern, revealing a statistically comparable rate of adequate response to the first antipsychotic medication among patients who later developed TRS (46.7%) [38]. This shared efficiency rate at the first pharmacological hurdle underscores a profound similarity in the therapeutic recalcitrance of both conditions, suggesting a common underlying pathophysiology of poor initial network responsivity.

This similarity extends beyond phenomenology to a potential shared mechanism of desperation: just as recurrent IEDs in TRE may reflect a network chronically poised at the brink of a seizure, the IEDs in TRS could be construed as the brain’s “desperate” attempt to trigger a convulsive event that might, paradoxically, serve a reset function akin to ECT. This perspective is powerfully supported by the long-observed clinical antagonism between epilepsy and psychosis, exemplified by the phenomenon of forced normalization or alternative psychosis, where suppression of seizures (and presumably *IEDs*) leads to the emergence of psychotic symptoms [65,66]. This reciprocal relationship suggests that paroxysmal neuronal synchronization and psychotic states may exist on a physiological continuum, where excessive inhibition of one precipitates the expression of the other. Consequently, the chronic IEDs in TRS might represent a maladaptive, low-grade “seizure-equivalent” process, stuck in a subconvulsive state that is insufficient to induce a therapeutic reset but potent enough to disrupt normal network function and perpetuate a psychotic milieu.

This hypothesis of a transition from a potential compensatory signal to a core perpetuating factor is intriguing but remains tentative. Longitudinal studies tracking EEG changes from the first episode onwards are essential to test whether IEDs precede or follow the development of treatment resistance. Thus, we hypothesize that IEDs undergo a transition from a potential failed compensatory signal to a core perpetuating factor in the neuropathology of TRS. Its original biological “purpose” is ultimately lost, overwhelmed by the chronicity and severity of the neurochemical dysregulation and locked into a pathological equilibrium reminiscent of the epilepsy-psychosis continuum.

In summary, our findings position EEG abnormalities—both IEDs and background slowing—as core neurophysiological features of treatment-resistant schizophrenia. The convergence of evidence from our cohort, the parallels with treatment-resistant epilepsy, and the emerging understanding of the kynurenine pathway collectively suggest that what we observe on EEG may be more than a mere marker of pathology. It may represent the electrophysiological signature of a brain caught in a vicious cycle of hyperexcitability, metabolic dysregulation, and failed homeostatic attempts. This perspective not only enriches our pathophysiological understanding but also highlights the potential of routine EEG as a clinically accessible tool for stratifying patients and guiding personalized treatment approaches.

### 4.4. Clinical Implications and Future Directions

Our findings have direct translational relevance. Routine EEG represents an accessible and cost-effective tool for stratifying patients with schizophrenia. The identification of IEDs, especially when coupled with moderate-to-severe background slowing, should alert clinicians to a high probability of treatment resistance. This EEG-defined subgroup may benefit from tailored therapeutic strategies, such as:

- Adjunctive mood stabilizers/anticonvulsants (e.g., valproate, lamotrigine) to dampen neuronal hyperexcitability, drawing on the parallel with epilepsy management.
- KP-modulating interventions, an area of active preclinical research aimed at reducing QUIN or enhancing KYNA signaling.
- Targeted neurostimulation (e.g., transcranial direct current stimulation—tDCS, or repetitive transcranial magnetic stimulation—rTMS) protocols designed to modulate cortical excitability and enhance slow-wave activity.

Future research must adopt a longitudinal design to track the evolution of EEG features alongside clinical progression and biochemical markers (e.g., KP metabolites in blood or CSF). Furthermore, interventional studies examining whether suppressing IEDs improves clinical outcomes in TRS are crucial for validating its causative role and for testing the hypothesis that IEDs are not merely a marker but a mechanistic driver of treatment resistance.

### 4.5. Study Limitations

This study has several limitations. Its cross-sectional design precludes causal inferences about the relationship between EEG features and the development of treatment resistance. Longitudinal studies are needed to determine whether the observed EEG abnormalities precede or follow the establishment of treatment resistance. The sample size, though substantial, requires replication in larger, independent cohorts. While we employed strict clinical criteria for TRS, the assessment was naturalistic. Direct measurement of KP metabolites or other inflammatory markers was not performed in conjunction with the EEG, a necessary step for future mechanistic studies. Fourth, we did not systematically control for the potential confounding effects of antipsychotic medication. Patients with TRS are frequently on higher doses, polypharmacy, or clozapine, all of which can influence EEG activity. While our groups were defined by treatment outcome, the medication regimen itself may have contributed to the observed EEG differences. Future studies should incorporate detailed pharmacokinetic and pharmacodynamic data to disentangle disease-related from treatment-related EEG changes.

Fifth, we did not perform sleep EEG recordings, which could increase the sensitivity for detecting IEDs [63]. It is well established in epilepsy that sleep potentiates the occurrence of interictal epileptiform discharges. In our patients, sleep EEG recordings were not feasible due to the severe mental state of some participants (presence of paranoid and hallucinatory experiences). Future studies could incorporate sleep deprivation or 24 h ambulatory EEG recordings to more accurately assess the prevalence of IEDs in TRS.

## 5. Conclusions

In conclusion, our study confirms that interictal epileptiform discharges and severe background slowing are key neurophysiological signatures associated with treatment-resistant schizophrenia. Moving beyond correlation, we propose an integrative hypothesis suggesting that IEDs might originate as a failed adaptive mechanism, potentially mirroring the brain’s mechanisms in treatment-resistant epilepsy. We hypothesize that in the enduring pathology of TRS, however, this activity may lose any beneficial purpose. It could become a maladaptive, subconvulsive “seizure-equivalent” process, trapped in a state that both reflects and contributes to a vicious cycle of excitotoxicity and network dysfunction—a state physiologically antagonistic to normalized conscious experience, as suggested by the epilepsy-psychosis continuum. Recognizing this potential duality enhances our pathophysiological understanding and opens novel avenues for stratification, suggesting that TRS patients with prominent IEDs might benefit from therapeutic strategies borrowed from the epilepsy armamentarium, aimed at modulating this aberrant excitatory drive towards a more balanced, homeostatic state. However, longitudinal and interventional studies are critically needed to test these hypotheses and establish causality.

## Figures and Tables

**Table 1:** Demographic and Clinical Characteristics of Patients with Treatment-Resistant Schizophrenia (TRS) and Those in Remission.

Characteristic	TRS Group (*n* = 39) Mean ± SD	Remission Group (*n* = 50) Mean ± SD	*p*-Value
Age (years)	36.82 ± 10.79	36.84 ± 10.26	0.994
Age of Onset (years)	23.33 ± 7.31	27.52 ± 8.45	0.018
Duration of Illness (years)	13.69 ± 11.45	9.14 ± 6.98	0.016
Height (cm)	169.79 ± 8.56	166.84 ± 7.45	0.073
Weight (kg)	75.00 ± 15.04	74.66 ± 16.24	0.919
Body Mass Index (kg/m^2^)	26.39 ± 4.86	26.88 ± 5.61	0.667

SD = Standard Deviation; indicates statistical significance (*p* < 0.05). *p*-values were derived from independent samples t-tests, except for Duration of Illness, where the Mann–Whitney U test was applied.

**Table 2:** Distribution of Background EEG Activity in the groups.

EEG Background Category	Remission Group (*n* = 50) *n* (%)	TRS Group (*n* = 39) *n* (%)
1. Normal	28 (56.0%)	4 (10.3%)
2. Mild Diffuse Slowing	15 (30.0%)	8 (20.5%)
3. Moderate Slowing	5 (10.0%)	19 (48.7%)
4. Severe Slowing	2 (4.0%)	8 (20.5%)
Median (IQR)	1.5 (1–2)	3.0 (3–4)

IQR = Interquartile Range. Group difference: Mann–Whitney U test, *p* < 0.001.

**Table 3:** Binary Logistic Regression Analysis: Independent Predictors of Treatment-Resistant Schizophrenia (TRS).

Predictor Variable	B	SE	Wald χ^2^	df	*p*-Value	Odds Ratio (OR)	95% CI for OR
IEDs (present vs. absent)	1.163	0.552	4.44	1	0.035	3.20	1.08–9.45
Background Severity Score	1.411	0.329	18.42	1	<0.001	4.10	2.15–7.82
Age of Onset (years)	−0.042	0.033	1.64	1	0.20	0.96	0.90–1.02
Illness Duration (years)	0.095	0.051	3.53	1	0.06	1.10	1.00–1.22
Gender (male vs. female)	0.712	0.495	2.07	1	0.15	2.04	0.77–5.38
Constant	−2.847	1.324	4.62	1	0.032	0.058	–

Model Summary: χ^2^(5) = 34.2, *p* < 0.001; Nagelkerke R^2^ = 0.42; Hosmer-Lemeshow goodness-of-fit: *p* = 0.67 Abbreviations: B = unstandardized regression coefficient; SE = standard error; df = degrees of freedom; CI = confidence interval; IEDs = interictal epileptiform discharges.

## Data Availability

The raw data supporting the conclusions of this article will be made available by the authors upon reasonable request.
